# The digital scribe in clinical practice: a scoping review and research agenda

**DOI:** 10.1038/s41746-021-00432-5

**Published:** 2021-03-26

**Authors:** Marieke M. van Buchem, Hileen Boosman, Martijn P. Bauer, Ilse M. J. Kant, Simone A. Cammel, Ewout W. Steyerberg

**Affiliations:** 1 Department of Information Technology & Digital Innovation, Leiden University Medical Center (LUMC), Leiden, The Netherlands; 2 CAIRELab, Leiden University Medical Center (LUMC), Leiden, The Netherlands; 3 Department of Quality & Patient Safety, Leiden University Medical Center (LUMC), Leiden, The Netherlands; 4 Department of Internal Medicine, Leiden University Medical Center (LUMC), Leiden, The Netherlands; 5 Department of Biomedical Data Sciences, Leiden University Medical Center (LUMC), Leiden, The Netherlands

**Keywords:** Health care, Computer science

## Abstract

The number of clinician burnouts is increasing and has been linked to a high administrative burden. Automatic speech recognition (ASR) and natural language processing (NLP) techniques may address this issue by creating the possibility of automating clinical documentation with a “digital scribe”. We reviewed the current status of the digital scribe in development towards clinical practice and present a scope for future research. We performed a literature search of four scientific databases (Medline, Web of Science, ACL, and Arxiv) and requested several companies that offer digital scribes to provide performance data. We included articles that described the use of models on clinical conversational data, either automatically or manually transcribed, to automate clinical documentation. Of 20 included articles, three described ASR models for clinical conversations. The other 17 articles presented models for entity extraction, classification, or summarization of clinical conversations. Two studies examined the system’s clinical validity and usability, while the other 18 studies only assessed their model’s technical validity on the specific NLP task. One company provided performance data. The most promising models use context-sensitive word embeddings in combination with attention-based neural networks. However, the studies on digital scribes only focus on technical validity, while companies offering digital scribes do not publish information on any of the research phases. Future research should focus on more extensive reporting, iteratively studying technical validity and clinical validity and usability, and investigating the clinical utility of digital scribes.

## Introduction

In the past few years, clinician burnout has become an acknowledged problem in healthcare. In a 2017 survey among 5000 US clinicians, 44% reported at least one symptom of burnout^1^. To investigate this problem, the National Academy of Medicine formed a committee focused on improving patient care by supporting clinician well-being. The committee’s extensive report, called Taking Action Against Clinician Burnout, describes reasons for clinician burnout. An important reason is the increasing administrative burden^2^. Since the introduction of the electronic health record (EHR), the time spent on administrative tasks has increased to approximately half of a clinician’s workday^3–5^. These administrative tasks decrease clinicians’ career satisfaction^6^ and negatively affect the clinician–patient relationship^7^. Other studies have assessed the relationship between EHR-use and burnout and found that more time spent on the EHR, especially after-hours, was linked to a higher risk of burnout^8,9^.

Recently, clinicians have hired medical scribes to reduce the administrative burden, i.e., persons who manage administrative tasks, such as summarizing a consultation. Studies show positive results for the use of medical scribes, with clinicians spending more face-to-face time with patients and less after-hour time on the EHR^10,11^. Although a medical scribe might seem like the perfect solution, it shifts the burden to other personnel. As a result, direct medical costs increase, while the administrative burden remains substantial. Two recent perspectives^12,13^ describe the need for a so-called digital scribe. This digital scribe uses techniques such as automatic speech recognition (ASR) and natural language processing (NLP) to automate (parts of) clinical documentation. The proposed structure for a digital scribe includes a microphone that records a conversation, an ASR system that transcribes this conversation, and a set of NLP models to extract or summarize relevant information and present it to the physician. The extracted information could, for instance, be used to create clinical notes, add billing codes, or use the extracted information for diagnosis support.

Companies like Google, Nuance, Amazon, and many startups are creating a digital scribe^14–16^. Although much needed, there are several concerns about implementing a digital scribe in healthcare. These relate to technical aspects such as the accuracy of current ASR systems for transcription of spontaneous speech^13^ and a digital scribe’s ability to extract all the essential information from a non-linear, fragmented conversation^13,17^. There are also concerns related to a digital scribe’s clinical utility, such as the effect on a physician’s workflow. Such concerns need to be addressed before digital scribes can be safely implemented in practice. More specifically, successful implementation of an artificial intelligence (AI) tool, such as a digital scribe, requires a thorough investigation of its suitability, technical validity, clinical validity and usability, and clinical utility (see Box 1). A scoping review of current evidence is needed to determine the current status of the digital scribe and to make recommendations for future research.

Box 1: Four research phases Suitability: The first step aims to create a clear overview of the problem and find a suitable solution. In the digital scribe field, the problem is the administrative burden. Deciding on a suitable solution (e.g., symptom list, summary) is the next step towards determining the required model’s output and a reliable ground truth^52^. When the problem and solution are clear, researchers can find a suitable dataset or collect data themselves. Researchers should also check if the dataset contains any unintended bias or underrepresented groups.

Technical validity: Next, various models may be created and the best performing model determined^55^. Apart from determining the model’s overall performance, researchers should assess in which situations the model performs well and in which it performs less adequately. This includes assessing if the model performs consistently across different patient groups, for example gender^56^. The data source, model, and context in which the model was tested should all be described transparently^50^. Sharing data and code help the community better understand the models and enables researchers to build on past work^52^.

Clinical validity and usability: Once the model passes the technical validation, the researchers should perform a qualitative evaluation of the output with the end-user. This evaluation has two goals: first, to evaluate whether the output makes sense and is clinically relevant; second, to evaluate how the output affects clinical practice. This includes the presentation of the output, the most appropriate timing, and the effect on end-users’ decision making^57^.

Clinical utility: In this last step, the researchers should prospectively study the model in clinical practice. First, the model might run in clinical practice without showing the output to the end-users. At specific time points, end-users analyze the output to identify any errors. If no new problems arise, a prospective study can be set up to determine clinical impact.

### Objective

The purpose of the present study is to perform a scoping review of the literature and contact companies on the current status of digital scribes in healthcare. The specific research questions are:

- Which methods are being used to develop (part of) a digital scribe? (Suitability)
- How accurate are these methods? (Technical validity)
- Have any of these methods been evaluated in clinical practice? (Clinical validity and usability, clinical utility)

## Methods

### Data search

We performed a scoping review based on the Preferred Reporting Items for Systematic Reviews and Meta-Analyses Extension for Scoping Reviews (PRISMA-ScR) statement^18^. We searched Medline, Web of Science, Arxiv, and ACL for all relevant articles until December 25, 2020. Furthermore, we scanned reference lists of relevant publications for additional articles. Search terms included terms describing the setting (clinical conversations) in combination with relevant methods (NLP, ASR) and usage of the output (clinical documentation). We also included “digital scribe” and “automated scribe” as search terms because these incorporate the setting, method, and goal. The full search queries can be found in Supplementary Table 1.

Besides, we aimed to include real-world data on existing digital scribes to bridge the gap between research and practice. Quiroz et al.^13^ provided a list of active companies in the digital scribe space: Robin Healthcare, DeepScribe, Saykara, Sopris Health, Amazon, Nuance. These companies were requested to provide unpublished performance data for their digital scribe.

### Inclusion and exclusion criteria

Our definition of a digital scribe is any system that uses a clinical conversation as input, either as audio or text, and automatically extracts information that can be used to generate an encounter note. We included articles that describe the performance of either ASR or NLP on clinical conversational data. A clinical conversation was defined as a conversation—in real life, over the phone, or via chat—between at least one patient and one healthcare professional. Because ASR and NLP are different fields of expertize and will often be described in separate studies, we chose to include studies that only focused on part of a digital scribe. Studies that described NLP models that were not aimed at creating an encounter note but, for example, extracted information for research purposes, were excluded. Articles written in any language other than English were excluded. Because of the rapidly evolving research field and the time lag for publications, proceedings were included.

### Study selection

Two reviewers (M.M.v.B. and S.A.C.) independently screened all articles on title and abstract, using the inclusion and exclusion criteria. The selected articles were assessed for eligibility by reading the full text.

### Data extraction and synthesis

The first reviewer extracted information from the included articles and the unpublished data provided by companies. The second reviewer verified the extracted information. The following aspects were extracted and assessed:

1. Setting and research phase
2. ASR models and performance
3. NLP tasks, models, and performance

## Results

### Study selection

**Fig. 1:**
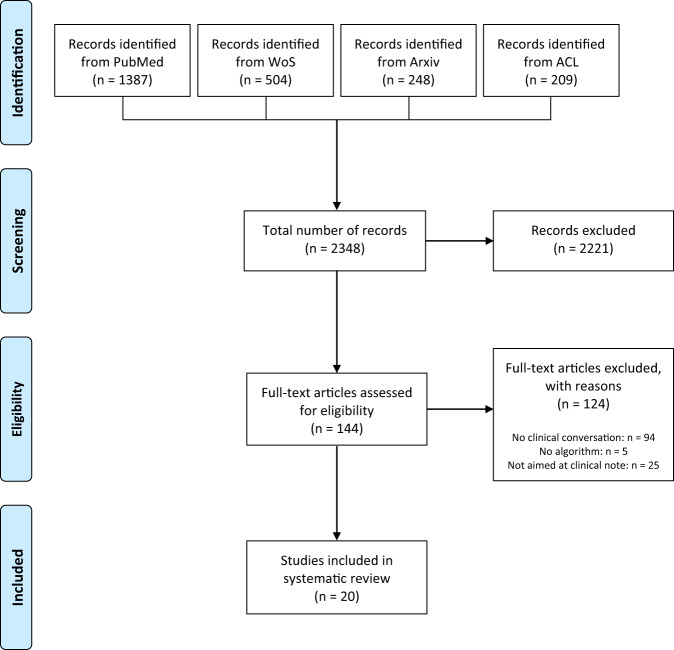
Inclusion flowchart. The four phases of article selection following the PRISMA-ScR statement.

Our search resulted in 2348 articles. After screening the titles and abstracts of these articles, we assessed 144 full-text articles for eligibility. We included 20 articles^19–38^ for our analysis (Fig. 1 and Supplementary Table 2). Of these, ten were conference proceedings^19–21,23,27,28,32,38^, seven were workshop proceedings^22,26,29,34–37^, two were journal articles^24,25^, and three were Arxiv preprints^30,31,33^.

Of the six contacted companies, DeepScribe^39^ was the only one to provide unpublished data on their digital scribe system’s performance. We were unable to obtain performance data from other companies.

### Setting and research phase

Although all 20 studies aimed to decrease the administrative burden of clinical documentation in some way, the specific approaches and the setting differed greatly among studies. Three studies focused on improving the ASR for clinical conversations as the first step towards accurately extracting information from them^19,21,36^. Eleven studies chose to manually transcribe the conversations and performed NLP tasks on the transcripts^20,22,24,25,27,30–32,34,35,40^. Five studies used input data representative of the input of an implemented digital scribe (ASR transcripts or chat dialogs)^26,28,33,37,38^.

Settings differed greatly between studies, as most did not define a specific specialty^19,21–23,26,31–36,38^, while others were focused on primary care^20,25,27^, home hemodialysis^24^, orthopedic encounters^37^, cardiology, family medicine, internal medicine^31^, and patient-clinician dialogs via a telemedicine platform^28^. Fifteen studies were performed by or in collaboration with a company^19–21,23,25–28,30,33–37^.

All included studies focused on the technical validity of the digital scribe; only two studies investigated the clinical validity and usability by performing a qualitative evaluation with end-users^24,28^. None of the studies investigated the clinical utility.

### Automatic speech recognition (ASR)

In total, seven of 20 studies used ASR to automate clinical documentation^19,21,23,26,33,36,38^, and one company provided data on their ASR system. Of these, two studies and the company presented a new ASR model^19,21^, four used ASR to transcribe conversations as input for NLP models^26,33,37,38^, one presented a model to correct ASR errors^36^, and one compared the performance of existing ASR systems on clinical conversations^23^ (see Supplementary Table 3).

In all studies, the metric used to evaluate the ASR transcripts was the word error rate (WER, see Box 2). The lowest WER was 14.1%, according to the unpublished data provided by DeepScribe. This ASR system combines Google Video Model^41^, IBM Watson^42^, and a custom-made Kaldi model^43^. The best performing published (as opposed to the unpublished data provided by DeepScribe) ASR system had a WER of 18%^19^. Four studies^23,26,33,36^ used existing ASR systems and found WERs between 38% (IBM Watson) and 65% (Mozilla DeepSpeech^44^).

One study^36^ presented a postprocessing model to correct ASR errors. By using an attention-based neural network, WERs were improved from 41 to 35% (Google Speech-to-Text^45^) and 36 to 35% (off-the-shelf open-source model^46^).

Box 2: Explanation of metrics WER: This metric counts the number of substitutions, deletions, and insertions in the automatic transcript, compared to the manual transcript. The lower the WER, the better the performance.

F1 score: The F1 score is the harmonic mean between the precision (or positive predictive value) and the recall (or sensitivity).

ROUGE: This is a score that measures the similarity between the automatic summary and the gold standard summary, in unigrams (ROUGE-1), bigrams (ROUGE-2), or the longest common subsequence (ROUGE-L). The ROUGE-L score considers sentence-level structure, while the ROUGE-1 and ROUGE-2 scores only examine if a uni- or bigram occurs in both the automatic and gold standard summary.

### Natural language processing (NLP) tasks and models

**Fig. 2:**
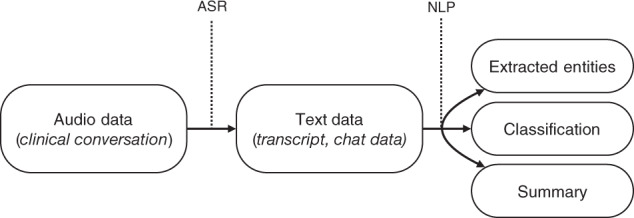
Overview of a digital scribe. Scope of the different aspects and techniques of the included digital scribes.

The NLP tasks that were performed could be split into three categories: entity extraction^20,25–27,30,32,35,38^, classification^22,24,30–35^, and summarization^22,24,28,29,31,37^ (see Fig. 2 and Supplementary Table 4). All except one study used word embeddings (see Box 3) as input to their model. This study did not use word embeddings as input but used a clustering model to create 2000 clusters^24^. The model’s input consisted of the current words’ clusters, the number of words, and the previous words’ clusters.

### Entity extraction

**Fig. 3:**
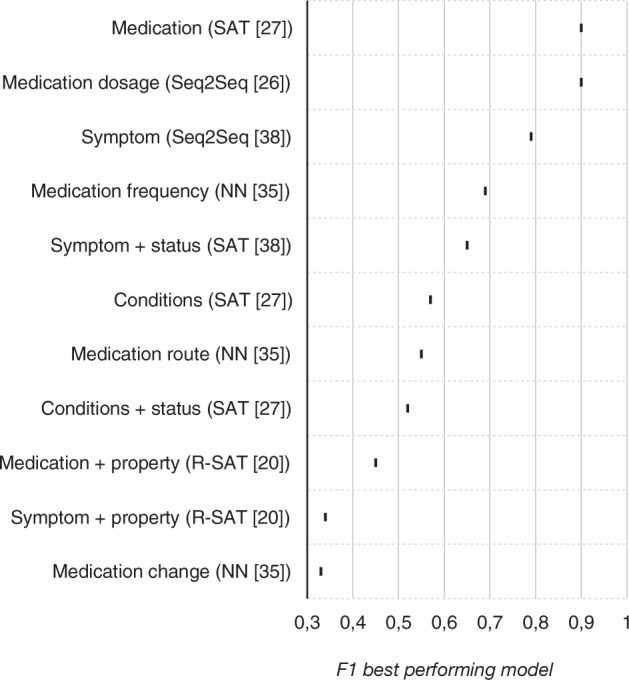
Performance of entity extraction models. Highest F1 scores per entity extraction task, with best performing model.

The eight studies using entity extraction focused on extracting symptoms^20,25,27,32,38^, medication regimen^20,26,27,32,35^, and conditions^27^. However, the studies differed in the combination of entities and properties they extracted. Several studies examined the possibility of extracting symptoms and identifying whether a symptom was present or not^20,27,38^, while only one study focused on all the other combinations (i.e., medication dosage, frequency, symptom properties). Almost all studies reported their results as F1 scores (see Box 2). The tasks of extracting the medication, medication dosage, and symptom resulted in the highest F1 scores and thus showed the best performance (see Fig. 3).

All studies used neural networks, although the type of neural network differed. Some studies used general neural networks^22,30,35^, but most used neural network-based sequence models with attention (see Box 3). In the studies that compared different types of models, the neural networks with attention layer achieved higher F1 scores than the neural networks without attention layer (see Fig. 3).

Three studies^27,32,38^ performed an error analysis of which one investigated the symptoms that were incorrectly labeled as “absent”. The authors reported that these symptoms were often discussed in multiple talk-turns. In the other study^27^, ten human annotators categorized the cause of all labeling errors and the impact on the clinical note. They concluded that 16 to 32% of the errors did not affect the clinical note’s content. Furthermore, most errors were caused by a failure of the model to take context into account or the lack of knowledge about a patient’s medical background. In 29 to 42% of the errors, the human annotators agreed with the model, showing the difficulty of annotating the data. One study reported that most errors originated from informal language use and describing symptoms in physical manifestations (“I only sleep for 4 h”)^32^.

Two studies^26,38^ made a comparison between manually transcribed and automatically transcribed data regarding the performance of their entity extraction model. Both found that models trained on manually transcribed data outperform the model trained on the automatically transcribed data. The difference in F1 for extracting symptoms was 0.79 versus 0.72, whereas the difference in ROUGE-1 (see Box 2) for extracting medication dosage was 85 versus 79^26^.

#### Classification

Six studies performed a type of classification^22,24,30,33–35^, which varied greatly: in which summary section it belonged^22,24,33,34^; if a sentence was said by the patient or the phycisian^33^; relevant diagnoses of the patient^22,30^; if any abnormalities were found in the medical history^30^ (see Supplementary Table 4). A greater variety of models was used for classification than for entity extraction, although neural networks were used most often. The classification tasks resulting in the highest F1 scores were the classification of primary diagnosis, utterance type, and entity status (see Supplementary Table 4). In two of these tasks, support vector machines were used.

One study^33^ tested their classification model on manually transcribed data and automatically transcribed data. The model performed better on the manually transcribed data, with a difference in F1 score ranging from 0.03 to 0.06, although they did not mention if the difference was significant.

One study assessed possible disparities of their classification model towards disadvantaged groups^34^. They formed 18 disadvantaged and advantaged groups based on gender, ethnicity, socioeconomic status, age, obesity, mental health, and location. In 7 of 90 cases, there was a statistically significant difference in favor of the advantaged group. The main reason for the disparity is a difference in the type of medical visit. For example, “blood” is a strong lexical cue to classify a sentence as important for the “Plan” section of the summary, but this word is said less often in conversations with Asian patients.

#### Summarization

Six studies^22,24,28,29,31,37^ used NLP to summarize the conversation between patient and healthcare professional automatically. Four studies used pointer generator networks to create a hybrid extractive and abstractive summary^28,29,31,37^. One of these studies approached the summarization problem as a machine translation problem, where the transcript has to be “translated” to a summary^37^. This study compared the pointer generator network to three other attention-based models (see Supplementary Table 4).

The other two studies used extractive methods, where the output of the classification or entity extraction models was used to extract the most important utterances from the conversation^22,24^. The combination of these utterances formed the summary. One of these studies did not compare their summaries to a gold standard^24^; the other study asked physicians to extract the most important utterances as gold standard^22^. The F1 score for the latter study was 0.61.

All studies using pointer generator networks reported their results as ROUGE-scores. However, one study only reported their results as ROUGE-L relative error rate reduction^37^, limiting the comparability with the other studies.

The ROUGE-L scores in the other three studies were 0.42^31^, 0.55^28^, and 0.55^29^. One study also presented a model that returned summaries with a ROUGE-L of 0.58, but this was based on manually extracting noteworthy utterances^31^. When using the same model with automatically extracted noteworthy utterances, the performance dropped to 0.42.

The best performing model used a pretrained pointer generator network (see Box 3) fine-tuned on medical dialog summarization, with an added penalty for the generator distribution to force the model to favor copying text from the transcript over generating new text^28^. The other models were: a topic-aware pointer-generator network using embeddings (see Box 3)^29^, which takes the topic of the current segment into account when copying or generating the next word; an LSTM architecture with BERT embeddings to extract noteworthy utterances (see Box 3)^31^; a combination of a transformer and pointer generator network that creates a summary per summary section (see Box 3)^37^.

Two studies included physicians to evaluate their summaries^24,28^. One study examined physicians’ ability to answer questions about patient care based on the automatic summary^24^. They did not find any significant difference in physicians’ answers using the human-made summaries compared to the automatic summaries. Another study asked physicians to rate the amount of relevant information in the summaries^28^. Physicians found that 80% of the summaries included “all” or “most” relevant facts. The study did not specify which parts were deemed relevant or not or if the model missed specific information.

DeepScribe did not provide information on the models used for summarization but included how often a summary needed to be adjusted in practice. They report that 77% of their summaries do not need modification by a medical scribe before being sent to the physician. Furthermore, 74% of their summaries do not need modification from a medical scribe or a physician before being accepted as part of the patient’s record, saving time on administrative tasks.

Box 3: Neural network-based sequence models with attention and word embeddings Attention-based neural networks: These models specifically take the sequence of the words into account, and have an attention layer. This layer acts as a filter, only passing the relevant subset of the input to the next layer.

- Sequence2sequence (seq2seq)^58^: the seq2seq model uses a bidirectional encoder LSTM to include context, and has an attention mechanism to focus on the relevant parts of the input.
- Span-attribute tagging model (SAT)^38^: the sat model extracts symptoms and classifies them as present or not. It first identifies the relevant parts of the text and then classifies those relevant parts into symptoms that are or are not present. The relation-span-attribute tagging model (R-SAT) is a variant of the SAT that focuses on relations between attributes.
- Pointer generator network (PGNet)^59^: PGNets are based on the seq2seq architecture. The added value of a PGNet is that it has the ability to generate new words or copy words from the text, increasing the summary’s accuracy.

Word embeddings: Word embeddings are used to numerically represent words in a way that similar words have similar representations. For example, the words “physician”, “clinician”, and “doctor” will have similar representations. There are different types of word embeddings, but the most important distinction for this review is between context-sentitive and context-insensitive embeddings. Context-sensitive embeddings have different representation for words that have multiple meanings. For example, the word “bank” can mean a riverbank, or a financial institution. Some word embeddings, like word2vec^60^, allow only one representation per word, whereas context-sensitive embeddings like ELMo^61^ and BERT^62^ can distinguish the different meanings of the word “bank”.

## Discussion

This scoping review provides an overview of the current state of the development, validation, and implementation of digital scribes. Although the digital scribe is still in an early research phase, there appears to be a substantial research body testing various techniques in different settings. The first results are promising: state-of-the-art models are trained on vast corpora of annotated clinical conversations. Although the performance of these models varies per task, the results give a clear view of which tasks and which models yield high performance. Reports of clinical validity and usability, and especially clinical utility are, however, mostly lacking.

All studies focusing on ASR used physician–patient dialogs without further specification of the setting. In general, existing ASR systems not explicitly trained on clinical conversations did not perform well, with WERs up to 65%. The speech recognition systems trained on thousands of clinical conversations had WERs as low as 18%. This WER is still high compared to the claimed WERs of general, state-of-the-art, available ASR systems that attain WERs as low as 5%^47^. The difference in performance can be explained by the uncontrolled setting of clinical conversations with background noise, multiple speakers, and the spontaneity of the speech^13^. However, these aspects were not reported by any of the studies, complicating the comparison of WERs. Two new approaches decreased the WER by postprocessing the automatic transcript^36^ and combining multiple ASR systems (DeepScribe). These approaches are promising new ways to decrease the WER. However, what is most important is whether the WER is good enough to extract all the relevant information. Currently, the NLP models trained on manually transcribed data outperform those trained on automatically transcribed data, which means there is room for improvement of the WER.

When comparing the different NLP tasks, the diverseness in both tasks and underlying models was large. The classification models focused mainly on extracting metadata, such as relevance or structure induction of an utterance, and used various models ranging from logistic regression to neural networks. The entity extraction models were more homogeneous in models but extracted many different entities, complicating the comparison, whereas the summarization task was mostly uniform, both in models and in metrics. One notable aspect of the NLP tasks overall is the use of word embeddings. Only one study did not use word embeddings, but this was a study from 2006 when context-sensitive word embeddings were not yet available. All the other studies were published after 2019 and used various word embeddings as input. The introduction of context-sensitive word embeddings has been essential for extracting entities and summarizing clinical conversations.

In the entity extraction task, the specific tasks, such as extracting symptoms, led to better performance than more general tasks, such as extracting symptoms and their properties. An explanation for this is the heterogeneity in, for example, symptom properties, which entail the location, severity, duration, and other characteristics of a symptom. These properties can be phrased in various ways, in contrast to medication or frequency, which will be much more homogeneous in phrasing. Therefore, this homogeneity leads to many more annotations per entity, increasing performance.

The same pattern was observed in the models, where the addition of an attention layer increased performance. This finding is in line with previous studies on neural attention^48,49^, which describe the decrease in neural networks’ performance with increased input length. By adding weights to the input text, the model knows which parts of the text are important for its task. Adding attention not only improves performance; it also decreases the amount of training data needed, which is useful in a field such as healthcare, where gathering large datasets can be challenging.

In the studies performing the entity extraction task, the error analyses showed that often, symptoms, medications, or properties are hard to interpret even by human annotators. This result is in line with the concerns discussed in the introduction, questioning if a model would accurately extract all relevant information from a non-linear, fragmented conversation. However, this takes the concern one step further, namely how the “gold standard” will be determined if there is ambiguity between human annotators. More research is needed to define methods for developing gold standards. Shafran et al.^27^ have taken an exciting first step towards such a method by publishing an article about the development of their corpus, including how they dealt with ambiguity and labeling errors.

The studies investigating summarization of the clinical conversation used both extractive and abstractive summarization techniques. However, the extractive techniques resulted in a list of the most important utterances instead of a new, full summary. Therefore, the studies performing abstractive summarization are more interesting to discuss. All four studies used the same model, the pointer generator network^28,29,31,37^. This network’s advantage, especially with the studies’ additions, makes sure it copies more words than it generates, keeping the summary as close to the conversation as possible. Two studies also included a quality check by physicians, which gives more insight into the possibility of implementation^24,28^. However, it would have been interesting to include error analyses to investigate the models’ blind spots.

### Future work

First of all, we believe it is vital to improve the ASR for clinical conversations further and use them as input for NLP models. A remarkable finding was that most studies used manually transcribed conversations as input to their NLP model. These manual transcripts may outperform automatically transcribed conversations regarding data quality, leading to an overestimation of the results. NLP models that require manual transcription may increase administrative burden when implemented in clinical practice.

Secondly, the current body of research is mostly focused on improving the performance of different models. Although some studies performed error analyses and qualitative analyses of the model’s output, most did not. Moreover, most studies did not fully cover the technical validity phase because of insufficient reporting on the setting, data, and situations in which the model succeeded and failed. This information is essential to describe for a model that could potentially be implemented in clinical practice. The proposed models might contain bias or lead to unintended results, as Ferracane and Konam^34,37^ show. This study is an inspirational example of how researchers can investigate the strengths and weaknesses of their model. A recent paper by Hernandez-Boussard et al.^50^ proposes reporting standards for AI in healthcare, which should be the basis for reporting on digital scribes as well.

Although most studies are in an early development phase, including qualitative analyses of the model’s output is necessary to know if the solution researchers or developers are working on is applicable in practice. The lack of implementation following the development of an AI model is common in healthcare^51^, which can be limited by investigating clinical validity and usability while working on technical validity. A good example is the study by Joshi et al.^28^, where physicians qualitatively analyze the model’s output. These results lead to new insights for improving technical validity. Studying these two research phases iteratively leads to a solution that is well-suited for clinical practice.

Most of the presented models need to be technically and clinically validated before moving on to the clinical utility phase. However, the companies already offering digital scribes seem to have skipped all four research phases, including clinical utility. We urge these companies to publish data on their digital scribes’ technical validity, clinical validity and usability, and clinical utility. Not only is transparency in the model and its performance crucial for clinical practice, but it also helps the community better understand the models and enables researchers to build on past work^52^.

The suitability phase falls outside the scope of this review but is nevertheless vital for developing and implementing the digital scribe. One research group has published several studies investigating which parts of a clinical conversation are relevant for creating a summary and how physicians see the potential role of a digital scribe^53,54^. These studies should be the starting point for researchers and developers working on a digital scribe.

### Strengths and limitations

The current work is the first effort to review all available literature on developing a digital scribe. We believe our search strategy was complete, leading to a comprehensive and focused scope of the digital scribe’s current research body. By adding the company’s data, we create a broader overview than just the digital scribe’s scientific status. However, this data is unpublished, which means we have to trust the company in providing us with legitimate data. We hope this review is an encouragement for other companies to study their digital scribes scientifically.

One limitation is the small amount of journal papers included in this review, as opposed to the amount of Arxiv preprints and workshop proceedings. These types of papers are often refereed very loosely. However, only including journal papers would not lead to a complete scope of this quickly evolving field.

Contacting various digital scribe companies was a first step towards gaining insight into implemented digital scribes and their performance on the different ASR and NLP tasks. Although only one company replied, we believe it is a valuable addition to this review. It indicates that their implemented digital scribe does not differ significantly in techniques or performance from the included studies’ models while already saving physicians’ time. Nevertheless, it highlights the gap between research and practice. The studies published by companies all describe techniques that are not part of a fully functional digital scribe (yet). However, none of the companies offering digital scribes have published about the technical validity, clinical validity and usability, or clinical utility of their systems.

## Conclusion

Although the digital scribe field has only recently started to accelerate, the presented techniques achieve promising results. The most promising models use context-sensitive word embeddings in combination with attention-based neural networks. However, the studies on digital scribes only focus on technical validity, while companies offering digital scribes do not publish on any of the research phases. Future research should focus on more extensive reporting, iteratively studying technical validity and clinical validity and usability, and investigating the clinical utility of digital scribes.

## Supplementary information

Supplementary Information

## Data Availability

Any data generated or analyzed are included in this article and the Supplementary Information files. Aggregate data analyzed in this study are available from the corresponding author on reasonable request.
