# Supplementary material for: The digital scribe in clinical practice: a scoping review and research agenda
Source: NPJ Digit Med. 2021 Mar 26;4:57. doi: 10.1038/s41746-021-00432-5 (PMC7997964; doi:10.1038/s41746-021-00432-5)
Supplement: Supplementary file 1 — Supplementary Information [file 41746_2021_432_MOESM1_ESM.pdf]

Supplementary Information

Supplementary Table 1: search queries per database.

Database	Query
Medline	(digital scribe[tiab] OR digital scribes[tiab] OR automated scribe[tiab] OR automated scribes[tiab]) OR ((conversat*[tiab] OR speech[tiab] OR transcri*[tiab] OR encounter*[tiab] OR dialog*[tiab] OR consultation*[tiab] OR interview*[tiab]) AND (natural language processing[ti] OR NLP[ti] OR "Machine Learning"[Mesh] OR machine learning[ti] OR artificial intelligence[ti] OR "AI"[ti] OR summar*[ti] OR knowledge extraction[ti] OR information retrieval[ti] OR natural language generation[ti] OR "Algorithms"[Mesh] OR Algorithm*[tw] OR "Speech Recognition Software"[Mesh] OR "Speech Recognition"[ti]) AND (documentation[tiab] OR "Medical Records"[Mesh] OR "record"[tiab] OR "records"[tiab] OR note[tiab] OR notes[tiab] OR "EHR"[tiab] OR "EPR"[tiab] OR chart*[tiab])) AND ("2005/01/01"[PDAT] : “3000/12/31”[PDAT])
arXiv	order: -announced_date_first; size: 50; page_start: 100; classification: Computer Science (cs); include_cross_list: True; terms: OR abstract=clinical conversation; OR abstract=clinical encounter; OR abstract=clinical consultation; OR abstract=medical conversation; OR abstract=medical encounter; OR abstract=medical consultation
Web of Science	(TS=("digital scribe" OR "digital scribes" OR "automated scribe" OR "automated scribes") OR (TS=(conversat* OR speech OR transcri* OR encounter* OR dialog* OR consultation* OR interview*) AND TI=(natural language processing OR NLP OR HealthNLP OR machine learning OR artificial intelligence OR "AI" OR summar* OR knowledge extraction OR information retrieval OR natural language generation OR Algorithm* OR "Speech Recognition")) AND TS=(documentation OR "record" OR "records" OR note OR notes OR "EHR" OR "EPR" OR chart*)) *AND* **LANGUAGE:** (English)
ACL	("clinical encounter" OR "medical encounter" OR “clinical conversation” OR “medical conversation”) AND ("automatic speech recognition" OR "natural language processing") AND (“documentation” OR “encounter note”) site:aclweb.org

Supplementary Table 2: Overview of included articles.

Author	Year	University/company	Title	Setting	Transcription	NLP task
Lacson, Barzilay, Long [29]	oct. 2006	Massachusetts Institute of Technology, USA	Automatic analysis of medical dialogue in the home hemodialysis domain: structure induction and summarization,	Home hemodialysis (phone calls)	Manual	Classification, summarization
Chiu et al.[24]	jun. 2018	Google Research, USA	Speech recognition for medical conversations	Patient-clinician dialogues (unspecified)	ASR (new model) and manual	-
Kodish-Wachs et al.[28]	dec. 2018	Cerner Corporation, USA	A systematic comparison of contemporary automatic speech recognition engines for conversational clinical speech	Patient-clinician dialogues (unspecified)	ASR (comparison of existing ASR systems)	-
Rajkomar et al. [30]	mar. 2019	Google Research, USA	Automatically Charting Symptoms from Patient-Physician Conversations Using Machine Learning,	Primary care	Manual	Entity extraction
Du et al.[43]	jun. 2019	Google Research, USA	Extracting Symptoms and their Status from Clinical Conversations	Primary care	Manual	Entity extraction
Shafey, Soltau, Shafran[26]	jul. 2019	Google Research, USA	Joint Speech Recognition and Speaker Diarization via Sequence Transduction	Patient-clinician dialogues (unspecified)	ASR (new model) and manual	-
Du et al.[25]	aug. 2019	Google Research, USA	Learning to Infer Entities, Properties and their Relations from Clinical Conversations	Patient-clinician dialogues (unspecified)	ASR (input for NLP tasks) and manual	Entity extraction
Liu, Lee, Chen[34]	oct. 2019	Institute for Infocomm Research, Singapore	Topic-Aware Pointer-Generator Networks for Summarizing Spoken Conversations	Nurse-patient dialogues	Manual	Summarization
Jeblee et al.[27]	nov. 2019	University of Toronto, Canada	Extracting relevant information from physician-patient dialogues for automated clinical note taking	Patient-clinician dialogues (unspecified)	Manual	Classification, summarization

Shafran et al.[32]	mar. 2020	Google Research, USA	The Medical Scribe: Corpus Development and Model Performance Analyses	Primary care	Manual	Entity extraction
Enarvi et al.[42]	jul. 2020	Nuance Communications, Microsoft Corporation, USA	Conversations Using Sequence-to-Sequence Models	Orthopedic encounters	ASR (input for NLP tasks)	Summarization
Krishna et al.[35]	jul. 2020	Carnegie Mellon University & Abridge AI	Extracting Structured Data from Physician-Patient Conversations by Predicting Noteworthy Utterances	Patient-clinician dialogues (unspecified)	Manual	Classification, entity extraction
Schloss, Konam[38]	jul. 2020	Abridge AI	Towards an Automated SOAP Note: Classifying Utterances from Medical Conversations	Patient-clinician dialogues (unspecified)	ASR (input for NLP tasks) and manual	Classification
Joshi et al. [33]	sep. 2020	Stanford University & Curai	Dr, Summarize: Global Summarization of Medical Dialogue by Exploiting Local Structures	Patient-clinician dialogues via telemedicine platform	N/A	Summarization
Krishna et al.[36]	oct. 2020	Carnegie Mellon University, USA	Generating SOAP Notes from Doctor-Patient Conversations	Cardiology, family medicine, internal medicine	Manual	Classification, summarization
DeepScribe (unpublished data)	oct. 2020	DeepScribe	-	Patient-clinician dialogues (unspecified)	ASR (new model)	Summarization
Khosla et al.[37]	oct. 2020	Carnegie Mellon University, USA	MedFilter: Improving Extraction of Taks-relevant Utterances through Intervention of Discourse Structure and Ontological Knowledge	Patient-clinician dialogues (unspecified)	Manual	Entity extraction
Mani, Palaskar, Konam[41]	oct. 2020	Carnegie Mellon University & Abridge AI	Towards Understanding ASR Error Correction for Medical Conversations	Patient-clinician dialogues (unspecified)	ASR (postprocessing)	-

Selvaraj, Konam[31]	oct. 2020	Abridge AI, USA	Medication Regimen Extraction from Medical Conversations	Patient-clinician dialogues (unspecified)	ASR (input for NLP tasks) and manual	Entity extraction
Patel, Konam, Selvaraj[40]	nov. 2020	University of Massachusetts & Abridge AI, USA	Weakly Supervised Medication Regimen Extraction from Medical Conversations	Patient-clinician dialogues (unspecified)	Manual	Classification, entity extraction
Ferracane, Konam[39]	dec. 2020	Abridge AI, USA	Towards Fairness in Classifying Medical Conversations into SOAP Sections	Patient-clinician dialogues (unspecified)	Manual	Classification

NLP: natural language processing, ASR: automatic speech recognition.

Supplementary Table 3: Overview of articles describing Automatic Speech Recognition.

Author	Dataset	ASR system/model	WER
Chiu et al.[24]	90,000 clinical conversations	RNN<br>LAS	20%<br>18%
Du et al.[43]	90,000 clinical conversations	Google Speech	20%
Shafey, Soltau, Shafran[26]	100,000 clinical conversations	RNN	19%
Kodish-Wachs et al.[28]	34 unscripted, simulated interviews	Bing Speech<br>Google Speech<br>IBM Watson Speech<br>Azure MAVIS<br>Nuance<br>SpeechAnywhere<br>Mozilla DeepSpeech	49%<br>44%<br>38%<br>41%<br>58%<br>65%
Selvaraj, Konam[31]	6,693 clinical conversations	IBM Watson Speech<br>Google Speech	±50%<br>±50%
Schloss, Konam[38]	8130 annotated transcripts	Google Video Model	40%
Mani, Palaskar, Konam[41]	3807 transcripts	Google Speech + seq2seq error correction<br>ASPIRE + seq2seq error correction	41% —> 35%<br>36% —> 35%
DeepScribe (unpublished)	100,000 clinical conversations	Combination of Google Video Model, IBM Watson, and own custom made Kaldi model.	14%

RNN: recurrent neural network, LAS: listen, attend and spell.

Supplementary Table 4: Overview of articles describing natural language tasks.

Author	Dataset	Input	Classification	Entity extraction	Summarization	Models	Performance metrics[2]
Du et al.[25] (2019)	92,000 unlabeled, transcribed clinical conversations, 2,950 labeled primary care conversations	Knowledge graph embeddings	-	Symptoms, medications, their properties and relations	-	R-SAT	F1 symptom + property: 0.34<br>F1 symptom + status: 0.57<br>F1 medication + property: 0.45
Du et al.[43] (2019)	90,000 unlabeled, transcribed clinical conversations, 2,950 labeled primary care conversations	Embeddings	-	Symptom and status	-	SAT<br>Seq2Seq	F1 symptom<br>SAT: 0.78<br>Seq2Seq: 0.79<br>F1 symptom + status<br>SAT: 0.65<br>Seq2Seq: 0.64
Enarvi et al.[42] (2020)	800,000 orthopedic encounters	Embeddings	-	-	Machine translation approach to summarization	RNN with attention, hierarchical encoder, PGNet, **transformer + PGNet**	ROUGE-L, HPI: 0.19<br>RES: 0.51<br>PE: 0.65<br>AP: 0.42
Ferracane, Konam[39] (2020)	63,000 annotated transcripts	ELMo embeddings	Utterance classification to SOAP section	-	-	Attention layer, biLSTM + LSTM	F1: 0.38
Jeblee et al.[27] (2019)	800 transcribed clinical conversations	ELMo embeddings	Utterance type, entity modality and pertinence, SOAP, primary diagnosis	Clinically relevant entities	List of entities with their attributes per SOAP section	SVM, NN, LR, RF, LDA	F1, utterance type: 0.71<br>Entity extraction: 0.64<br>Attribute classification: 0.77<br>Entity modality: 0.62<br>Entity pertinence: 0.60<br>SOAP: 0.60<br>Primary diagnosis: 0.78
Joshi et al. [33] (2020)	25,000 chat conversations, 3000 summarized snippets	Embeddings	-	-	Hybrid extractive and abstractive summarization	PGNet	ROUGE-L: 0.55
Khosla et al.[37] (2020)	7,000 annotated transcripts	BERT embeddings	-	Symptoms, medication, and complaint extraction.	-	Multi-speaker biLSTM + ontological knowledge, hierarchical loss function, positional info	F1 (macro average), symptom extraction: 0.36<br>Medication extraction: 0.36<br>Condition extraction: 0.11
Krishna et al.[35] (2020)	Patient-clinician dialogues (unspecified)	BoW + tf-idf (baseline), BERT embeddings	Relevant diagnoses, RoS abnormalities (present or not)	-	-	LR, SVC, NB, RF, GB, **NN**	F1 (macro average), diagnosis prediction: 0.57<br>RoS abnormality prediction: 0.40
Krishna et al.[36] (2020)	7,000 annotated transcripts	Embeddings, BERT embeddings	-	-	Utterance relevance, SOAP classification, abstractive summarization per section	PGNet, LR, biLSTM	ROUGE-L: 0.58

Lacson, Barzilay, Long [29] (2006)	118 transcribed phone conversations	Lexical features, metadata, contextual features per talk turn	Structure induction	-	First and longest dialogue turn per structure type	Boosting classifier, clustering algorithm	Accuracy classification: 73% F1 summary: 0.61
Liu, Lee, Chen[34] (2019)	100,000	Glove embeddings	-	-	Hybrid extractive and abstractive summarization	Seq2Seq, **PGNet**	ROUGE-L: 0.55
Patel, Konam, Selvaraj[40] (2020)	63,000 annotated transcripts	BERT embedding + attention score	Attributes (frequency, route, change)	Medication regimen	-	NN	F1, extraction: 0.52 Classification: 0.53
Rajkomar et al. [30] (2019)	90,000 transcribed clinical conversations, 2,547 annotated primary care conversations	Not specified	-	Symptoms	-	RNN	Sensitivity to identify symptoms: 67.7% Symptoms clearly mentioned: 48.8% Sensitivity of clearly mentioned symptoms: 92.2%.
Schloss, Konam[38] (2020)	8,130 annotated transcripts	BoW (baseline), ELMo embeddings	Utterance classification to SOAP section & speaker role	-	-	MC, NB, LR, RF, DLB + attention layer + biLSTM + LSTM	F1, SOAP classification: 0.48 Speaker classification: 0.59
Selvaraj, Konam[31] (2020)	6,693 transcribed clinical conversations	ELMo, BERT & ClinicalBERT embeddings	-	Medication, dosage and frequency	-	Seq2Seq	F1 dosage: 0.90 F1 frequency: 0.46
Shafran et al.[32] (2020)	6,000 transcribed primary care and internal medicine conversations	Not specified	-	Symptoms, medication, and conditions, including their properties	-	R-SAT	F1, symptoms: 0.72 Conditions: 0.57 Medications: 0.90. Symptoms + status: 0.60, Conditions + status: 0.52.

[1]When multiple models are compared, the best performing model is shown in bold.

[2]When multiple models are compared, the performance metrics of the best performing model are shown. Lexical features: unigrams, bigrams (as word clusters), metadata: length of talk turn, contextual features: previous talk turn. + explanation different models. SAT: span-attribute tagging, Seq2Seq: sequence-to-sequence, R-SAT: relation-span-attribute tagging, L-LDA: labeled-Latent Dirichlet allocation, NB: naïve Bayes, SVM: support vector machines, CRF: conditional random fields, DT: decision tree, RF: random forest, NN: neural network, LR: logistic regression, RNN: recurrent neural network, tf-idf: term frequency – inverse document frequency.
